# Diagnosis Rates of Chronic Hepatitis B in Privately Insured Patients in the United States

**DOI:** 10.1001/jamanetworkopen.2020.1844

**Published:** 2020-04-09

**Authors:** Eiichi Ogawa, Yee Hui Yeo, Nolan Dang, Michael H. Le, Donghak Jeong, Sally Tran, Linda Henry, Ramsey Cheung, Mindie H. Nguyen

**Affiliations:** 1 Division of Gastroenterology and Hepatology, Department of Medicine, Stanford University Medical Center, Palo Alto, California; 2 Department of General Internal Medicine, Kyushu University Hospital, Fukuoka, Japan; 3 Division of Gastroenterology and Hepatology, Veterans Affairs Palo Alto Health Care System, Palo Alto, California

## Abstract

**Importance:**

To achieve the World Health Organization goal of viral hepatitis elimination by 2030, it is important to estimate current rates of chronic hepatitis B (CHB) diagnosis and treatment.

**Objective:**

To provide an accurate accounting of the number of patients with CHB aged 6 years or older who have not yet been diagnosed in the United States.

**Design, Setting, and Participants:**

This cross-sectional study used the commercial US Truven Health MarketScan Database (138 634 154 privately insured individuals in January 2007 to December 2014) to identify patients with CHB diagnosis and the National Health and Nutrition Examination Survey to estimate the actual number of privately insured persons with CHB. Based on sex and age distribution derived from the US Census Bureau, we calculated the total population with CHB and the proportion of those who remained undiagnosed among the 198 073 302 privately insured individuals. Next, we identified diagnosed CHB patients who received 1 or more prescription for CHB medications to calculate the treatment rate for those with severe disease states, such as cirrhosis and hepatocellular carcinoma, that would warrant treatment. Analyses were performed from October 2017 to January 2020.

**Main Outcomes and Measures:**

The rate and number of patients with CHB who remained undiagnosed and treatment rates for patients with CHB who have cirrhosis or hepatocellular carcinoma.

**Results:**

Among the 198 073 302 privately insured individuals (48.55% male; 15.52% aged 6-17 years; 84.48% aged ≥18 years), there were 511 029 (95% CI, 317 733-704 325) individuals with CHB, but only 95 075 of these had been diagnosed, yielding a diagnosis rate of only 18.60% (95% CI, 13.50%-29.92%), meaning that 81.40% (95% CI, 70.08%-86.50%) were undiagnosed. The treatment rates were 34.79% (95% CI, 33.31%-36.27%) for those with cirrhosis and 48.64% (95% CI, 45.59%-51.69%) for those with hepatocellular carcinoma.

**Conclusions and Relevance:**

In this study, only approximately 1 in 5 privately insured patients with CHB had been diagnosed. Only one-third of patients with CHB who had cirrhosis and one-half who had hepatocellular carcinoma received antiviral therapy. Further efforts are needed to improve the current situation of poor connection to care for patients with CHB, especially for those with advanced liver disease.

## Key Points

**Question:**

What is the actual rate of diagnosis for patients with chronic hepatitis B in the US?

**Findings:**

This cross-sectional study using data on more than 100 million patients from a large US nationwide claims database of patients with private insurance and the National Health and Nutrition Examination Survey found that only 18.60% of privately insured patients with chronic hepatitis B had been diagnosed.

**Meaning:**

This study found low rates of hepatitis B diagnosis among privately insured individuals, suggesting that barriers to diagnosis may be both financial and nonfinancial; additional research is needed to characterize these barriers and to develop interventions to improve diagnosis rates.

## Teaser

This cross-sectional study compares the prevalence of diagnosis with chronic hepatitis B infection from a large database of privately insured patients with the prevalence of infection found in the National Health and Nutrition Examination Survey to estimate the prevalence of undiagnosed chronic hepatitis B in the US.

## Introduction

As of 2015, the World Health Organization (WHO) estimated that despite an effective vaccine that provides 98% to 100% protection against the hepatitis B virus (HBV), an estimated 292 million people were still living with chronic hepatitis B infection (CHB) worldwide.^1^ Left untreated, CHB can progress to cirrhosis and hepatocellular carcinoma (HCC).^2^ In fact, HBV-related HCC causes at least 54% of all liver cancer worldwide, and the most common risk factor for liver cancer is CHB.^3^ Those with CHB have a 25% to 40% lifetime risk of developing liver cancer.^4^ However, the number of people aware of having CHB is strikingly low. In 2016, the WHO suggested that only 10.5% (27 million) of those with CHB were aware of their illness; and of those, only 16.5% (4.5 million) were receiving treatment.^5^

Concurrent with efforts to curb the incidence of HCC, the WHO has initiated a global strategy to eliminate infectious hepatitis by the year 2030. The impetus for this strategy is a combination of the availability of curative treatments for the hepatitis C virus, preventive vaccination for HBV, and effective viral suppressive drugs for those with CHB. Therefore, to help curb the growing incidence of HCC and to assist in the development of strategies for the elimination of viral hepatitis by 2030, it is important to have an accurate accounting of the number of patients infected with HBV in the US, as low awareness and treatment rates for CHB are not just limited to low-resource countries. Thus, the aim of this study was to use nationwide databases to estimate the number of patients who remain undiagnosed with HBV in the US, so that plans can be developed to target those most at risk for HBV.

## Methods

### Study Design and Data Sources

This study was conducted in accordance to the 1975 Declaration of Helsinki,^6^ and the study protocol was approved by the institutional review board at Stanford University. Written informed consent was obtained from all NHANES participants. Reporting followed the Strengthening the Reporting of Observational Studies in Epidemiology (STROBE) reporting guideline. Study data were analyzed from October 2017 to January 2020.

Our study included patients aged 6 years or older in all data sources and calculations. First, we identified patients with CHB diagnosis using one of the largest private insurance databases in the US, the Truven Health MarketScan Research Database (Truven), which is housed within the Population Health Science Center at Stanford University, Palo Alto, California. Truven is a large national administrative claims database with data for 138 634 154 individuals with private health insurance coverage between January 2007 and December 2014. First, we determined the number of patients with at least 1 inpatient or 2 outpatient diagnoses of CHB (based on *International Classification of Diseases, Ninth Revision, Clinical Modification [ICD-9-CM]* code of 070.22, 070.23, 070.32, or 070.33). We then calculated the total number of patients with CHB diagnosis for the entire US population with private insurance based on the age- and sex-standardized prevalence of patients with CHB diagnosis using age distribution data obtained from 2014 population data from the US Census Bureau 2012-2016 American Community Survey.^7^

Next, we identified participants from the National Health and Nutrition Examination Survey (NHANES) from 2007 to 2014 who reported having private insurance and tested positive for hepatitis B surface antigen (HBsAg) to calculate the true prevalence of CHB in this population (for the purpose of this study, CHB was defined as testing positive for HBsAg, since the prevalence of acute hepatitis B is likely extremely low). The NHANES is a population-based database of the US civilian population comprising serial surveys that use a complex, multistage sampling method to represent the noninstitutionalized general US population. We also calculated the age- and sex-standardized diagnosed and undiagnosed prevalence of CHB for this population and the total CHB disease burden for the US population with private insurance using age distribution data obtained from 2014 population data from the US Census Bureau American Community Survey for 2012 to 2016.^7^

Further detailed information on the Truven, NHANES, and US Census Bureau databases is available in the eAppendix in the Supplement.

### Study Outcomes 

The first main outcome of this study was the rate and number of patients with CHB who remained undiagnosed. We further examined treatment rates for patients with CHB who had cirrhosis or hepatocellular carcinoma.

### Statistical Analysis

We calculated the number of patients with CHB who were not yet diagnosed as the difference between the number of privately insured US patients who had been diagnosed with CHB from the Truven database and the total number of patients who should have been diagnosed from the NHANES database. We also calculated the percentage of patients with CHB diagnosis and the percentage of patients who had not yet been diagnosed. We calculated the 95% confidence intervals for these estimates.

Next, as part of a cohort substudy, we followed up patients diagnosed with CHB in the Truven database to identify those who received treatment during the study follow-up period to calculate CHB treatment rates. We chose to use only outpatient medications because data on medications during hospitalization are limited in the Truven database. In addition, CHB treatment with nucleos(t)ide analogue is a long-term process for almost all patients with CHB. Therefore, even if initiated in the inpatient setting, it should have been continued as an outpatient. We defined treatment as having at least 1 prescription of interferon-alfa, pegylated interferon, lamivudine, adefovir, telbivudine, entecavir, tenofovir disoproxil fumarate, emtricitabine/tenofovir disoproxil fumarate, or tenofovir alafenamide at any time during the study period. Instead of depending on the laboratory values to determine eligibility criteria for antiviral therapy, we estimated treatment rate in patients with advanced disease, such as cirrhosis or HCC, since most if not all of these patients should have met criteria for therapies. We performed univariate and multivariable logistic regression to identify factors associated with antiviral treatment for this cohort.

In addition, we performed sensitivity analysis to include patients with CHB diagnosis in the Truven database using only 1 inpatient or 1 outpatient (vs 1 inpatient or 2 outpatient) encounters to define CHB diagnosis. This sensitivity analysis would provide the higher estimate for the number of patients with CHB diagnosis and a lower percentage of patients with CHB who were not yet diagnosed. All statistical tests were 2-tailed, and *P* < .05 was considered statistically significant.

## Results

### Number of Patients With CHB Diagnosis 

From the Truven database, we identified 138 634 154 individuals (48.55% male) who were privately insured and were aged 6 years or older (20.57% aged 6-17 years; 79.43% aged ≥18 years). From this cohort, we determined that a total of 63 133 patients had a CHB diagnosis (1 inpatient or 2 outpatient encounters with a CHB diagnosis code). Table 1 shows the number of patients with CHB diagnosis stratified by age, sex, insurance types, and US region. As a sensitivity analysis, we determined the number of patients with CHB diagnosis based on 1 inpatient or 1 outpatient (vs 1 inpatient or 2 outpatient) encounters to be 97 701, showing the number of patients stratified by age, sex, insurance type, and US region. eTable 1 and eTable 2 in the Supplement provide the age- and sex-standardized CHB prevalence data for the US population of 198 073 302 privately insured persons with CHB diagnoses based on 1 inpatient or 2 vs 1 outpatient encounter with a CHB diagnosis, respectively. The corresponding age- and sex-standardized prevalence of patients with CHB diagnosis were 0.0480% and 0.0743%, yielding respective totals of 95 075 and 147 168 persons with known CHB diagnosis, respectively.

**Table 1.** Number of Privately Insured Patients With a CHB Diagnosis^a^

Subgroup	Individuals, No.	1 Inpatient or 2 Outpatient		1 Inpatient or 1 Outpatient	
		Patients With CHB Diagnosis, No.	Diagnosed CHB Prevalence, % (95% CI)	Patients With CHB Diagnosis, No.	Diagnosed CHB Prevalence, % (95% CI)
Overall	138 634 154	63 133	0.0455 (0.0452-0.0459)	97 701	0.0705 (0.0700-0.0709)
Age group, y					
6-17	28 520 831	808	0.0028 (0.0026-0.0030)	1586	0.0056 (0.0053-0.0058)
18-34	39 866 980	13 199	0.0331 (0.0325-0.0337)	20 848	0.0523 (0.0516-0.0530)
35-44	22 576 301	16 693	0.0739 (0.0728-0.0751)	24 913	0.1104 (0.1090-0.1117)
45-54	22 678 600	17 212	0.0759 (0.0748-0.0770)	26 522	0.1169 (0.1155-0.1184)
55-64	17 038 737	12 086	0.0709 (0.0697-0.0722)	19 039	0.1117 (0.1102-0.1133)
≥65	7 952 705	3135	0.0394 (0.0380-0.0408)	4793	0.0603 (0.0586-0.0620)
Sex					
Male	67 312 312	36 604	0.0544 (0.0538-0.0549)	55 311	0.0822 (0.0815-0.0829)
Female	71 321 842	26 529	0.0372 (0.0367-0.0376)	42 390	0.0594 (0.0589-0.0600)
Insurance plan					
Preferred provider organization	87 512 508	34 013	0.0389 (0.0385-0.0393)	53 605	0.0613 (0.0607-0.0618)
Health maintenance organization	17 372 574	15 426	0.0888 (0.0874-0.0902)	22 582	0.1300 (0.1283-0.1317)
Other	33 749 072	13 694	0.0406 (0.0399-0.0910)	21 514	0.0637 (0.0629-0.0646)
Region					
Northeast	24 686 367	15 569	0.0631 (0.0621-0.0641)	25 375	0.1028 (0.1015-0.1041)
North Central	30 796 745	7332	0.0238 (0.0233-0.0244)	11 606	0.0377 (0.0370-0.0384)
South	52 785 069	19 283	0.0365 (0.0360-0.0370)	30 657	0.0581 (0.0574-0.0587)
West	25 568 826	30 063	0.1176 (0.1162-0.1189)	30 063	0.1176 (0.1162-0.1189)

Abbreviation: CHB, chronic hepatitis B.

^a^ Diagnosis was based on 1 inpatient, 1 outpatient, or 2 outpatient CHB diagnoses in the Truven database.

### Total CHB Disease Burden (Diagnosed and Undiagnosed)

Using CHB prevalence data from NHANES database, we estimated the age- and sex-standardized CHB prevalence for the US population of 198 073 302 persons with private insurance (48.55% male; 15.52% aged 6-17 years; 84.48% aged ≥18 years) to be 0.258% (95% CI, 0.160%-0.356%) and the total CHB disease burden for this population to be 511 029 (95% CI, 317 733-704 325) infected persons (Figure). In addition, as shown in eTable 3 in the Supplement, the distributions of age and sex among the Truven and privately insured NHANES populations were similar.

**Figure.**
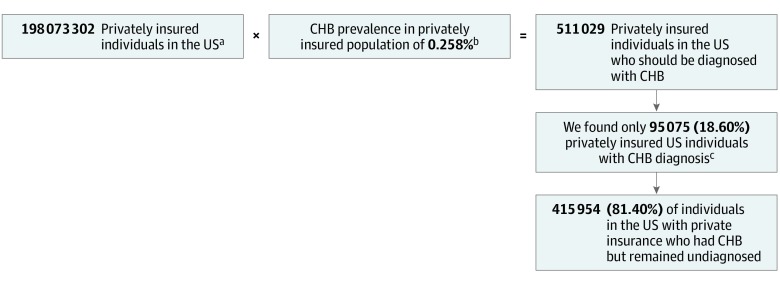
Graphical Study Overview and Summary CHB indicates chronic hepatitis B. ^a^Based on US Census Bureau data. ^b^Based on National Health and Nutrition Examination Survey data. ^c^Based on Truven/US Census Bureau data. Age- and sex-standardized prevalence of patients with CHB diagnosis as derived from Truven was 0.0480%, yielding a total number of patients diagnosed with CHB in the population of 198 073 302 privately insured individuals.

### Diagnosed and Undiagnosed Patients With CHB 

Of the total number of 511 029 privately insured individuals who we estimated should have been diagnosed with CHB (Figure), only 95 075 (18.60%; 95% CI, 13.50%-29.92%) had been diagnosed, leaving a total of 415 954 (81.40%; 95% CI, 70.08%-86.50%) remaining undiagnosed.

If using the number of individuals diagnosed with CHB (n = 147 168) from the sensitivity analysis that only required 1 inpatient or 1 outpatient diagnosis for CHB, the number of those with undiagnosed CHB would be 363 861 (511 029 − 147 168), giving a rate of undiagnosed CHB of 71.20% (95% CI, 53.68%-79.11%). The rate of diagnosed CHB remained low at 28.80% (95% CI, 20.89%-46.32%).

### Treatment Rates for CHB and Factors Associated With Treatment

Among the patients in the Truven database with CHB diagnosis, we found that 30.66% (95% CI, 30.28%-31.03%) had received at least 1 prescription for HBV medication at any time during the study period. Given a diagnosis rate of 18.60% for CHB, the estimated treatment rate for all CHB (diagnosed and undiagnosed) was only 5.70% (95% CI, 5.63%-5.77%).

As shown in Table 2, treatment rates were significantly higher in patients with cirrhosis (34.79%; 95% CI, 33.31%-36.27%) or HCC (48.64%; 95% CI, 45.59%-51.69%) (*P* < .001), significantly higher for those with gastroenterology or infectious disease care (35.64%; 95% CI, 35.08%-36.20%) compared with those followed up in a primary care clinic only (23.33%; 95% CI, 22.76%-23.90%) (*P* < .001), and significantly higher for those with health maintenance organization (HMO) insurance (33.32%; 95% CI, 32.54%-34.10%) compared with preferred provider organization insurance (30.37%; 95% CI, 29.86%-30.88%) (*P* < .001). Notably, we found the highest treatment rates among those with the lowest and highest out-of-pocket medical expense.

**Table 2.** Antiviral Treatment Rates in Privately Insured Patients With CHB Diagnosis^a^

Subgroup	Patients With CHB Diagnosis, No.	Patients Receiving Treatment, No.	Treatment Rate Among Patients With CHB Diagnosis, % (95% CI)	*P* Value
Overall	57 847	17 734	30.66 (30.28-31.03)	
Age group, y				
6-17	711	94	13.22 (10.73-15.71)	<.001
18-34	11 519	3298	28.63 (27.81-29.46)	
35-44	15 837	4958	31.31 (30.58-32.03)	
45-54	15 749	5110	32.45 (31.72-33.18)	
55-64	11 285	3542	31.39 (30.53-32.24)	
≥65	2746	732	26.66 (25.00-28.31)	
Sex				
Male	33 644	11 915	35.41 (34.90-35.93)	<.001
Female	24 203	5819	24.04 (23.50-24.58)	
Insurance plan				
Preferred provider organization	31 126	9454	30.37 (29.86-30.88)	<.001
Health maintenance organization	14 044	4679	33.32 (32.54-34.10)	
Other	12 677	3601	28.41 (27.62-29.19)	
Region				
Northeast	14 131	3968	28.08 (27.34-28.82)	<.001
North Central	6556	2110	32.18 (31.05-33.32)	
South	17 381	5765	33.17 (32.47-33.87)	
West	19 779	5891	29.78 (29.15-30.42)	
Type of clinician				
Primary care physician	20 937	4885	23.33 (22.76-23.90)	<.001
Gastrointestinal or infectious disease specialist	27 866	9931	35.64 (35.08-36.20)	
Other	9044	2918	32.26 (31.30-33.23)	
Out-of-pocket expense				
Quartile 1 (low)	16 852	5700	33.82 (33.11-34.54)	<.001
Quartile 2	14 358	3253	22.66 (21.97-23.34)	
Quartile 3	13 643	4202	30.80 (30.03-31.57)	
Quartile 4 (high)	12 994	4579	35.24 (34.42-36.06)	
Liver disease severity				
Noncirrhosis	52 853	15 854	30.00 (29.61-30.39)	<.001
Cirrhosis	3964	1379	34.79 (33.31-36.27)	
Hepatocellular carcinoma	1030	501	48.64 (45.59-51.69)	

Abbreviation: CHB, chronic hepatitis B.

^a^ Based on 2 outpatient encounters in the Truven database.

In multivariable analyses (Table 3), we found that factors associated with higher treatment rate included male sex (adjusted odds ratio [OR], 1.69; 95% CI, 1.62-1.75), HMO insurance (vs preferred provider organization: adjusted OR, 1.38; 95% CI, 1.32-1.48), gastrointestinal or infectious disease specialist care (vs primary care: adjusted OR, 1.82; 95% CI, 1.75-1.90), cirrhosis (adjusted OR, 1.12; 95% CI, 1.04-1.20), and HCC (adjusted OR, 1.93; 95% CI, 1.70-2.19). In addition, the second quartile of out-of-pocket expense level was associated with a 44% decrease in the likelihood of receiving therapy (adjusted OR, 0.56; 95% CI, 0.53-0.59) compared with those in the first quartile, followed by those in the third quartile (adjusted OR, 0.86; 95% CI, 0.82-0.91).

**Table 3.** Factors Associated With Antiviral Treatment for Privately Insured Patients With Chronic Hepatitis B Diagnosis

Factor	Univariate Analysis		Multivariable Analysis	
	Odds Ratio (95% CI)	*P* Value	Adjusted Odds Ratio (95% CI)^a^	*P* Value
Age group, y				
18-34	1 [Reference]		1 [Reference]	
6-17	0.38 (0.30-0.47)	<.001	0.40 (0.32-0.50)	<.001
35-44	1.14 (1.08-1.20)	<.001	1.10 (1.04-1.16)	<.001
45-54	1.20 (1.14-1.26)	<.001	1.11 (1.04-1.16)	<.001
55-64	1.14 (1.08-1.21)	<.001	1.01 (0.95-1.06)	.79
≥65	0.90 (0.83-0.99)	.04	0.75 (0.68-0.83)	<.001
Sex				
Female	1 [Reference]		1 [Reference]	
Male	1.73 (1.67-1.80)	<.001	1.69 (1.62-1.75)	<.001
Insurance plan				
Preferred provider organization	1 [Reference]		1 [Reference]	
Health maintenance organization	1.14 (1.09-1.19)	<.001	1.38 (1.32-1.48)	<.001
Other	0.91 (0.86-0.95)	<.001	0.93 (0.88-0.97)	.003
Region				
Northeast	1 [Reference]		1 [Reference]	
North Central	1.21 (1.14-1.29)	<.001	1.21 (1.13-1.29)	<.001
South	1.27 (1.21-1.33)	<.001	1.18 (1.12-1.24)	<.001
West	1.08 (1.03-1.14)	<.001	1.15 (1.09-1.21)	<.001
Type of clinician				
Primary care physician	1 [Reference]		1 [Reference]	
Gastrointestinal or infectious disease specialist	1.82 (1.74-1.89)	<.001	1.82 (1.75-1.90)	<.001
Other	1.56 (1.48-1.65)	<.001	1.45 (1.37-1.53)	<.001
Out-of-pocket expense				
Quartile 1 (low)	1 [Reference]		1 [Reference]	
Quartile 2	0.57 (0.54-0.60)	<.001	0.56 (0.53-0.59)	<.001
Quartile 3	0.87 (0.83-0.91)	<.001	0.86 (0.82-0.91)	<.001
Quartile 4 (high)	1.06 (1.01-1.11)	.01	1.08 (1.02-1.13)	.003
Liver disease severity				
Noncirrhosis	1 [Reference]		1 [Reference]	
Cirrhosis	1.24 (1.16-1.33)	<.001	1.12 (1.04-1.20)	.001
Hepatocellular carcinoma	2.21 (1.95-2.50)	<.001	1.93 (1.70-2.19)	<.001

^a^ Adjusted for age group, sex, insurance plan, region, type of clinician, out-of-pocket expense, and liver disease severity.

Sensitivity analysis estimating treatment rates for diagnosed CHB based on 1 outpatient visit (vs 2 outpatient visits as done in the main analysis) and regression analysis for this cohort showed similar results (eTable 4 and eTable 5 in the Supplement).

## Discussion

In our analysis of approximately 198 million people with private health insurance in the US, we found a poor care cascade for CHB, with less than 20% of patients with CHB having been diagnosed and only 6% having received treatment. Only 1 of every 3 patients with cirrhosis received treatment, and only 1 of every 2 patients with HCC were treated. This is very concerning because antiviral therapy is well known to decrease further disease progression among patients with cirrhosis, to improve survival among those with HCC, and to be cost-effective, especially when started at an early stage.^8,9,10^ Additionally, given that the whole study population had reasonably good health insurance coverage, these data suggest that the observed suboptimal connection to care may be due to other factors not directly related to medical care cost, such as cultural beliefs and lack of knowledge on the part of patients or clinicians.

The diagnosis rate from our study may be either underestimated or overestimated for various reasons, such as coding inaccuracies inherent in claims databases. A patient may also have been diagnosed prior to being entered into the Truven databases but never received care for CHB, so no CHB claims were recorded during the study period. However, this is probably unlikely given the length of the study period (2007-2014). Possibly, this result may be a better reflection of the lack of linkage to care for patients already diagnosed with CHB who are not receiving regular follow-up care as recommended by major US professional guidelines.^11,12^ In addition, our sensitivity analysis, which required only 1 outpatient encounter with CHB diagnosis (vs 2), confirmed this with a similarly low diagnosis rate.

The disparities by insurance types and levels of out-of-pocket expense are also notable. In our multivariable analysis, we found patients from HMOs were more likely to be diagnosed and treated, which is probably due to HMOs providing more standardized care. In reference to the out-of-pocket medical expenses, there was a U-shaped relationship between out-of-pocket expense and treatment rate. The lowest and the highest out-of-pocket expenses were associated with greater likelihood of receiving treatment. For those with the greatest expenses, we surmise that they were more likely to receive treatment as a result of potentially having more than 1 comorbidity, which necessitated more encounters with a practitioner and thus a better chance for CHB diagnosis and treatment. As such, further study is needed to determine the characteristics of HMOs that are associated with higher diagnosis and treatment rates.

When we compared the care provided by a primary care clinician to care received from gastrointestinal or infectious disease specialists, we found a higher treatment rate in patients also cared for by the specialists, even after adjustment for other demographic and medical factors, a finding in line with other reports.^13^ The reasons for this may be associated with both clinician and patient factors, which include clinician and patient knowledge and a higher index of suspicion of the presence of CHB among specialists, despite the social stigma and patient cultural beliefs associated with CHB diagnosis.^14,15,16,17^ Therefore, additional efforts must be directed toward both patient and primary health care clinician education, patient counseling, and a public campaign to end social and workplace discrimination against patients with CHB. These interventions are especially important given the fact that our study population included only privately insured patients in the US, who are generally considered to have better coverage than those with government insurance or no insurance, so those most at risk for CHB may need broader outreach efforts to improve their connection to appropriate HBV care.

The immigration patterns to the US may also have affected the CHB diagnosis rate and treatment. We found that the prevalence of diagnosed CHB in was the lowest for the pediatric group and increased with age, a finding consistent with the epidemiology of CHB in the US, where CHB is positively mitigated by HBV vaccination practices but negatively mitigated by current immigration patterns.^18,19,20,21^ This is further supported by the recently reported overall steady prevalence of CHB over the past decade, rather than a decrease, which is likely due to the consistent rate of immigration from highly endemic areas, such as Eastern Asia and sub-Saharan Africa, as indicated by the highest prevalence of CHB occurring in the western US, an area where many immigrants from Asia settle.^19,22,23,24^

The diagnosis and treatment rates estimated in our study were also notably lower than the HBV awareness and treatment rate (about one-third) reported in NHANES participants in prior studies.^25,26^ However, this difference may not be so unexpected for a number of reasons. First, NHANES participants with HBV may not be as representative of patients with HBV as those in the Truven population. The NHANES uses systematic sampling methods to conduct lab testing and complex surveys that can severely affect the likelihood of participation among many participants at high risk for HBV, such as immigrants, due to language and social and/or cultural barriers. Second, survey participants tend to be more motivated, which could bias toward higher disease diagnosis, awareness, and treatment rates. Third, our current study ascertained treatment by objective identification of anti-HBV prescriptions, while the NHANES data were based on patient report, which can be subject to recall bias and mistaken identification of other medications for anti-HBV medications. We also acknowledge that the presence of an HBV prescription does not necessarily mean the patient actually takes the prescribed medication.

### Limitations

This study has limitations. First, we lacked race/ethnicity^27^ and laboratory data, so we were unable to determine the impact of these variables on the diagnosis and treatment eligibility for CHB. Nonetheless, we did find that among patients who would be expected to be treated (eg, most patients with cirrhosis or HCC), only 34% with cirrhosis and 48% with HCC were treated. Second, we were unable to account for patients without private insurance, including those with government or no insurance, those incarcerated, or marginalized populations such as individuals who use intravenous drugs. However, we believe that the diagnosis and treatment rates for CHB would be even lower if these populations were included, which further highlights the importance of our current study results. In addition, CHB prevalence estimates from NHANES were based on the presence of only 1 HBsAg test and can include patients with acute hepatitis B. However, this number is likely very small, as acute hepatitis B is rare, especially in the NHANES population.

## Conclusions

From this large, nationwide cross-sectional study among approximately 198 million people aged 6 years and older with private insurance coverage in the US, we estimated that less than 20% of the more than 500 000 patients with CHB have actually been diagnosed, leaving more than 400 000 patients (80%) with undiagnosed CHB. The treatment rate was also very low, with less than 50% among those with cirrhosis or HCC receiving treatment for their CHB. Further efforts are urgently needed to improve the current situation of poor screening, diagnosis, and linkage to care for CHB in the US. Future studies must focus not only on the cost of medical care but other cultural and educational barriers to care. Additional research should focus not only on barriers to care but also the interventions that can be implemented to overcome these barriers.

## Notes

Supplement. **eTable 1.** Age- and Sex-Standardized Prevalence of Patients With Chronic Hepatitis B (CHB) Diagnosis in the Privately Insured Population in the United States With CHB Diagnosis Based on One Inpatient or Two Outpatient CHB Diagnosis **eTable 2.** Age- and Sex-Standardized Prevalence of Patients With Chronic Hepatitis B (CHB) Diagnosis in the Privately Insured Population in the United States With CHB Diagnosis Based on One Inpatient or One Outpatient CHB Diagnosis **eTable 3.** Demographic Characteristics Among Truven and NHANES Privately Insured Population (2007-2014) **eTable 4.** Antiviral Treatment Rates in Privately Insured Patients With Chronic Hepatitis B (CHB) Diagnosis Based on Only One Outpatient Encounter With CHB Diagnosis in the Truven Database **eTable 5.** Factors Associated With Antiviral Treatment Among Privately Insured Patients With Chronic Hepatitis B (CHB) Diagnosis Based on One Outpatient Encounter With CHB Diagnosis **eAppendix.** Additional Information of Data Sources
