# Supplementary material for: Diagnosis Rates of Chronic Hepatitis B in Privately Insured Patients in the United States
Source: JAMA Netw Open. 2020 Apr 9;3(4):e201844. doi: 10.1001/jamanetworkopen.2020.1844 (PMC7146097; doi:10.1001/jamanetworkopen.2020.1844)
Supplement: Supplement. — eTable 1. Age- and Sex-Standardized Prevalence of Patients With Chronic Hepatitis B (CHB) Diagnosis in the Privately Insured Population in the United States With CHB Diagnosis Based on One Inpatient or Two Outpatient CHB Diagnosis eTable 2. Age- and Sex-Standardized Prevalence of Patients With Chronic Hepatitis B (CHB) Diagnosis in the Privately Insured Population in the United States With CHB Diagnosis Based on One Inpatient or One Outpatient CHB Diagnosis eTable 3. Demographic Characteristics Among Truven and NHANES Privately Insured Population (2007-2014) eTable 4. Antiviral Treatment Rates in Privately Insured Patients With Chronic Hepatitis B (CHB) Diagnosis Based on Only One Outpatient Encounter With CHB Diagnosis in the Truven Database eTable 5. Factors Associated With Antiviral Treatment Among Privately Insured Patients With Chronic Hepatitis B (CHB) Diagnosis Based on One Outpatient Encounter With CHB Diagnosis eAppendix. Additional Information of Data Sources [file jamanetwopen-3-e201844-s001.pdf]

Supplementary Online Content

Ogawa E, Yeo YH, Dang N, et al. Diagnosis rates of chronic hepatitis B in privately insured patients in the United States. *JAMA Netw Open*. 2020;3(4):e201844. doi:10.1001/jamanetworkopen.2020.1844

eTable 1. Age- and Sex-Standardized Prevalence of Patients With Chronic Hepatitis B (CHB) Diagnosis in the Privately Insured Population in the United States With CHB Diagnosis Based on One Inpatient or Two Outpatient CHB Diagnosis
eTable 2. Age- and Sex-Standardized Prevalence of Patients With Chronic Hepatitis B (CHB) Diagnosis in the Privately Insured Population in the United States With CHB Diagnosis Based on One Inpatient or One Outpatient CHB Diagnosis
eTable 3. Demographic Characteristics Among Truven and NHANES Privately Insured Population (2007-2014)
eTable 4. Antiviral Treatment Rates in Privately Insured Patients With Chronic Hepatitis B (CHB) Diagnosis Based on Only One Outpatient Encounter With CHB Diagnosis in the Truven Database
eTable 5. Factors Associated With Antiviral Treatment Among Privately Insured Patients With Chronic Hepatitis B (CHB) Diagnosis Based on One Outpatient Encounter With CHB Diagnosis
eAppendix. Additional Information of Data Sources

This supplementary material has been provided by the authors to give readers additional information about their work.

© 2020 Ogawa E et al. *JAMA Network Open*.

eTable 1. Age- and sex-standardized prevalence of patients with chronic hepatitis B (CHB) diagnosis in the privately insured population in the United States with CHB diagnosis based on one inpatient or two outpatient CHB diagnosis

US Census Bureau and Truven		No. of patients in Truven	No. of patients with CHB diagnosis in Truven	Crude prevalence of patients with CHB diagnosis in Truven (%)	No. of privately insured patients in the US*	Age distribution of standard population*	Sex- and age-standardized prevalence of patient with CHB diagnosis (%)
**Overall**		**138,634,154**	**63,133**	**0.0455**	**198,073,302**	**1**	**0.0480**
**Male**							
**Age group (years)**	***6-17***	14,563,925	325	0.0022	15,716,620	0.079347	**0.0002**
	18-34	19,378,778	6,288	0.0324	23,752,206	0.119916	**0.0039**
	35-44	10,949,356	9,777	0.0893	14,033,352	0.070849	**0.0063**
	45-54	10,818,481	10,747	0.0993	15,598,419	0.078751	**0.0078**
	55-64	8,090,757	7,574	0.0936	14,473,187	0.073070	**0.0068**
	65 and older	3,511,015	1,893	0.0539	13,255,349	0.066921	**0.0036**
**Female**							
**Age group (years)**	***6-17***	13,956,906	483	0.0035	15,028,061	0.075871	**0.0003**
	18-34	20,488,202	6,911	0.0337	23,407,755	0.118177	**0.0040**
	35-44	11,626,945	6,916	0.0595	14,398,187	0.072691	**0.0043**
	45-54	11,860,119	6,465	0.0545	16,395,979	0.082777	**0.0045**
	55-64	8,947,980	4,512	0.0504	15,707,298	0.079300	**0.0040**
	65 and older	4,441,690	1,242	0.0280	16,306,890	0.082328	**0.0023**

*Data obtained from the US Census Bureau

© 2020 Ogawa E et al. *JAMA Network Open.*

eTable 2. Age- and sex-standardized prevalence of patients with chronic hepatitis B (CHB) diagnosis in the privately insured population in the United States with CHB diagnosis based on one inpatient or one outpatient CHB diagnosis

US Census Bureau and Truven		No. of patients in Truven	No. of diagnosed CHB in Truven	Crude diagnosed CHB prevalence in Truven (%)	No. of privately insured patients in the US	Age distribution of standard population*	Sex- and age-standardized diagnosed CHB prevalence (%)
**Overall**		**138,634,154**	**97,701**	**0.0705**	**198,073,302**	**1**	**0.0743**
**Male**							
**Age group (years)**	***6-17***	14,563,925	688	0.0047	15,716,620	0.079347	**0.0004**
	18-34	19,378,778	9,797	0.0506	23,752,206	0.119916	**0.0061**
	35-44	10,949,356	14,182	0.1295	14,033,352	0.070849	**0.0092**
	45-54	10,818,481	16,043	0.1483	15,598,419	0.078751	**0.0117**
	55-64	8,090,757	11,767	0.1454	14,473,187	0.073070	**0.0106**
	65 and older	3,511,015	2,834	0.0807	13,255,349	0.066921	**0.0054**
**Female**							
**Age group (years)**	***6-17***	13,956,906	898	0.0064	15,028,061	0.075871	**0.0005**
	18-34	20,488,202	11,051	0.0539	23,407,755	0.118177	**0.0064**
	35-44	11,626,945	10,731	0.0923	14,398,187	0.072691	**0.0067**
	45-54	11,860,119	10,479	0.0884	16,395,979	0.082777	**0.0073**
	55-64	8,947,980	7,272	0.0813	15,707,298	0.079300	**0.0064**
	65 and older	4,441,690	1,959	0.0441	16,306,890	0.082328	**0.0036**

© 2020 Ogawa E et al. *JAMA Network Open.*

eTable 3. Demographic characteristics among Truven and NHANES privately insured population (2007-2014)

		Truven	NHANES
		**No. of population (%)**	**No. of population (%)**
**Subgroup**	**Overall**	**138,634,154**	**16,580**
**Age group (years)**	***6-17***	28,520,831 (20.57)	3,990 (16.38)
	18-34	39,866,980 (28.76)	3,250 (22.20)
	35-44	22,576,301 (16.29)	2,172 (15.58)
	45-54	22,678,600 (16.36)	2,175 (17.33)
	55-64	17,038,737 (12.29)	2,140 (14.81)
	65 and older	7,952,705 (5.74)	2,853 (13.71)
**Sex**	***Male***	67,312,312 (48.55)	8,160 (48.38)
	Female	71,321,842 (51.45)	8,420 (51.62)

© 2020 Ogawa E et al. *JAMA Network Open.*

eTable 4. Antiviral treatment rates in privately insured patients with chronic hepatitis B (CHB) diagnosis based on only one outpatient encounter with CHB diagnosis in the Truven database

		No. of patients with CHB diagnosis	No. of treated CHB patients	Treatment rate among patients with CHB diagnosis (%, 95%CI)	*P*-value
**Subgroup**	**Overall**	**93,304**	**20,612**	**22.09 (21.83-22.36)**	
**Age group (years)**	***6-17***	1,308	108	8.26 (6.76-9.76)	<.001
	18-34	18,789	3,785	20.14 (19.57-20.72)	
	35-44	23,981	5,673	23.66 (23.12-24.19)	
	45-54	24,957	5,960	23.88 (23.35-24.41)	
	55-64	19,408	4,212	21.70 (21.12-22.28)	
	65 and older	4,861	874	17.98 (16.90-19.06)	
**Sex**	***Male***	52,866	13,936	26.36 (25.99-26.74)	<.001
	Female	40,438	6,676	16.51 (16.15-16.87)	
**Insurance plan**	***PPO***	51,145	11,060	21.62 (21.27-21.98)	<.001
	HMO	21,108	5,311	25.16 (24.58-25.75)	
	Other	21,051	4,241	20.15 (19.60-20.69)	
**Region**	***North East***	24,159	4,724	19.55 (19.05-20.05)	<.001
	North Central	10,921	2,509	22.99 (22.19-23.76)	
	South	29,105	6,769	23.26 (22.77-23.74)	
	West	29,128	6,610	22.69 (22.21-23.17)	
**Care provider specialty**	***PCP***	37,334	5,790	15.51 (15.14-15.88)	<.001
	GI/ID	40,243	11,378	28.27 (27.83-28.71)	
	Other	15,727	3,444	21.90 (21.25-22.55)	
**Out-of-pocket expense***	***Quartile 1 (low)***	23,326	6,198	26.57 (26.00-27.14)	<.001
	Quartile 2	23,326	3,707	15.89 (15.42-16.36)	
	Quartile 3	23,326	4,952	21.23 (20.70-21.75)	
	Quartile 4 (high)	23,326	5,755	24.67 (24.12-25.23)	

© 2020 Ogawa E et al. *JAMA Network Open.*

**Liver disease severity**	***Non-cirrhosis***	83,971	18,186	21.66 (21.38-21.94)	<.001
	Cirrhosis	7,827	1,821	23.27 (22.33-24.20)	
	HCC	1,506	605	40.17 (37.70-42.65)	

* Pre-CHB index diagnosis date

CI, confidence interval; PPO, preferred provider organization; HMO, health maintenance organization; PCP, primary care physician; GI/ID, gastrointestinal/infectious disease; HCC, hepatocellular carcinoma.

© 2020 Ogawa E et al. *JAMA Network Open*.

eTable 5. Factors associated with antiviral treatment among privately insured patients with chronic hepatitis B (CHB) diagnosis based on one outpatient encounter with CHB diagnosis

**Factors**		**Univariate analysis**		**Multivariable analysis**	
		**Odds ratio (95%CI)**	***P* value**	**Adjusted odds ratio (95%CI)**	***P* value**
**Age group (years)**	***18-34***	Referent		Referent	
	6-17	0.35 (0.29-0.43)	<.001	0.36 (0.29-0.44)	<.001
	35-44	1.22 (1.17-1.28)	<.001	1.17 (1.12-1.23)	<.001
	45-54	1.24 (1.18-1.30)	<.001	1.12 (1.07-1.17)	<.001
	55-64	1.09 (1.04-1.15)	<.001	0.96 (0.91-1.01)	.17
	65 and older	0.86 (0.80-0.94)	<.001	0.72 (0.66-0.78)	<.001
**Sex**	***Female***	Referent		Referent	
	Male	1.81 (1.75-1.87)	<.001	1.76 (1.71-1.82)	<.001
**Insurance plan**	***PPO***	Referent		Referent	
	HMO	1.21 (1.17-1.26)	<.001	1.45 (1.39-1.51)	<.001
	Other	0.91 (0.87-0.95)	<.001	0.93 (0.90-0.97)	.002
**Region**	***North East***	Referent		Referent	
	North Central	1.22 (1.16-1.29)	<.001	1.27 (1.20-1.34)	<.001
	South	1.24 (1.19-1.30)	<.001	1.16 (1.11-1.21)	<.001
	West	1.20 (1.15-1.25)	<.001	1.31 (1.25-1.37)	<.001
**Care provider specialty**	***PCP***	Referent		Referent	
	GI/ID	2.14 (2.07-2.22)	<.001	2.20 (2.12-2.29)	<.001
	Other	1.52 (1.45-1.60)	<.001	1.38 (1.31-1.45)	<.001
**Out-of-pocket expense***	***Quartile 1 (low)***	Referent		Referent	
	Quartile 2	0.52 (0.49-0.54)	<.001	0.51 (0.49-0.53)	<.001
	Quartile 3	0.74 (0.71-0.77)	<.001	0.75 (0.71-0.78)	<.001
	Quartile 4 (high)	0.90 (0.86-0.94)	<.001	0.93 (0.89-0.97)	.004
**Liver disease severity**	***Non-cirrhosis***	Referent		Referent	
	Cirrhosis	1.90 (1.03-1.15)	.001	0.98 (0.93-1.04)	.60

© 2020 Ogawa E et al. *JAMA Network Open.*

	HCC	2.42 (2.18-2.69)	<.001	2.04 (1.83-2.27)	<.001

CI, confidence interval; PPO, preferred provider organization; HMO, health maintenance organization; PCP, primary care physician; GI/ID, gastrointestinal/infectious disease; HCC, hepatocellular carcinoma.

© 2020 Ogawa E et al. *JAMA Network Open*.

eAppendix. Additional information of data sources

Truven Health MarketScan Research Databases

Data used for the estimation of the prevalence of diagnosed HBV were obtained from the Truven Health MarketScan® 2007 to 2014 Commercial and Medicare Supplemental Databases. These databases represent the health services of approximately 138 million employees, dependents, early retirees Consolidated Omnibus Budget Reconciliation Act (COBRA) continues, and Medicare-eligible retirees with employer-provided Medicare Supplemental plans in the U.S. with primary or Medicare supplemental coverage through privately insured fee-for-service, point-of-service, or capitated health plans. It includes private-sector health data from approximately 350 payers with more than 20 billion service records.

All enrollment, demographic records, utilization and expenditures of inpatient, outpatient, ancillary, and drug claims were recorded. The Commercial and Medicare Supplemental Databases are generally representative of the privately insured population in the U.S.

National Health and Nutrition Examination Survey (NHANES)

Data used for the estimation of the prevalence of HBV among the insured population were obtained from the National Health and Nutrition Examination Survey (NHANES) from 2007 to 2014. The NHANES database is a series of nutritional surveys, examinations, and laboratory tests collected from a sample of persons representative of the noninstitutionalized, civilian United States population. NHANES is approved by the institutional review board of the Centers for Disease Control and Prevention (CDC) and all participants provided written informed consent prior to participation in the survey. All NHANES data were deidentified and made publicly available by the National Center for Health Statistics of the CDC (https://www.cdc.gov/nchs/nhanes/index.htm).

NHANES tests blood specimens for hepatitis B surface antigen (HBsAg). Those who were positive for HBsAg were regarded to have current or past HBV infection. Serum specimens were processed, stored, and shipped to the Division of Viral Hepatitis, National Center for HIV/AIDS, Viral Hepatitis, STD, and TB Prevention, Centers for Disease Control and Prevention. From 2007-2014, HBsAg was tested through the use of the VITROS HBsAg

© 2020 Ogawa E et al. *JAMA Network Open.*

Reagent Pack and VITROS Immunodiagnostic Products HBsAg Calibrator on the Vitros ECI/ECiO Immunodiagnostic System (Ortho Clinical Diagnostics, Raritan, NJ). Additional detailed specimen collection and processing isntructions can be found in the NHANES Laboratory/Medical Technologists Procedures Manual, available at the NHANES website.

U.S. Census Bureau

We used resident population data for 2014 derived from the 2011-2015 American Community Survey 5-year Estimates to estimate by age and sex for both total and diagnosed CHB populations. U.S. Census Bureau is the federal government's largest statistical agency. This survey is a method of collecting and analyzing social, economic, and geographic data which provides information about the conditions of the United States, states, and counties. Every year, the Census Bureau publishes population estimates and demographic components of change, such as births, deaths, and migration. This data can be sorted by characteristics such as age, sex, and race, as well as by national, state, and county location.

US census data can be obtained online at website (https://www.census.gov), and then we investigated the total number of participants with private insurance in the U.S. according to the sex and age (https://factfinder.census.gov/faces/tableservices/jsf/pages/productview.xhtml?pid=ACS_17_5YR_B27002&prodType=table).

© 2020 Ogawa E et al. *JAMA Network Open.*
